# Potent Inhibition of Acid Ceramidase by Novel B-13 Analogues

**DOI:** 10.1155/2011/971618

**Published:** 2010-12-09

**Authors:** Denny Proksch, Jan Jasper Klein, Christoph Arenz

**Affiliations:** Institut für Chemie, Humboldt Universität zu Berlin, Brook-Taylor-Str 2, 12489 Berlin, Germany

## Abstract

The lipid-signalling molecule ceramide is known to induce apoptosis in a variety of cell types. Inhibition of the lysosomal acid ceramidase can increase cellular ceramide levels and thus induce apoptosis. Indeed, inhibitors of acid ceramidase have been reported to induce cell death and to display potentiating effects to classical radio- or chemo therapy in a number of *in vitro* and *in vivo* cancer models. The most potent *in vitro* inhibitor of acid ceramidase, B-13, recently revealed to be virtually inactive towards lysosomal acid ceramidase in living cells. In contrast, a number of weakly basic B-13 analogues have been shown to accumulate in the acidic compartments of living cells and to efficiently inhibit lysosomal acid ceramidase. However, introduction of weakly basic groups at the *ω*-position of the fatty acid moiety of B-13 led to a significant reduction of potency towards acid ceramidase from cellular extracts. Herein, we report a novel B-13-derived scaffold for more effective inhibitors of acid ceramidase. Furthermore, we provide hints for an introduction of basic functional groups at an alternative site of the B-13 scaffold that do not interfere with acid ceramidase inhibition *in vitro*.

## 1. Introduction

Besides their function as constituents of cellular membranes, sphingolipids play an important role as signalling molecules. They have essential functions for cell growth, cell differentiation, and cell death (apoptosis), thus being indispensable for cell homeostasis and normal cell development. Especially the equilibrium between ceramide and sphingosine-1-phosphate (S1P) seems to be decisive for a final cellular response [1–4]. The cellular “sphingolipid rheostat” is primarily regulated by three classes of enzymes: the ceramide generating sphingomyelinases, the ceramide degrading ceramidases, and finally the sphingosine kinases. Further enzymes controlling ceramide levels are the sphingomyelin synthases [5–7] and the glucocerebroside synthase [8]. 

Ceramide often accumulates after stimulation of cells with stress factors like inflammatory cytokines, UV radiation, X-rays, heat shock, and oxidative stress. As a consequence to such primary triggers, neutral or acid sphingomyelinases (nSMase or aSMase) are activated, in which both cleave sphingomyelin to form ceramide [9, 10]. The exact mechanism, by which the respective SMases are activated, is still not completely understood. Small molecule inhibitors that specifically act on the various sphingolipid-degrading enzymes can help in clarifying the underlying mechanisms. Recently, we developed a number of potent and specific inhibitors of the acid sphingomyelinase, which inhibited dexamethasone-induced apoptosis in HEK-293 cells, respectively [11–13]. The action of SMases, however, controls only the influx of ceramide and thus only partially contributes to understanding cellular ceramide concentration. The contribution coming from the degradation of ceramide by ceramidases (CDases) is similarly important. 

Several different CDases exist, which can be characterized by their different pH-optima (e.g., neutral [14] and alkaline [15]) and subcellular localization. The acid ceramidase (aCDase) is doubtlessly indispensable for the cellular ceramide degradation, and a deficiency in acid ceramidase leads to Morbus Farber, a recessively inherited lysosomal storage disease characterized by massive lysosomal ceramide accumulation. Patients have a strongly limited life expectancy and—depending on the residual enzyme activity—die within a few years. The phenotype of the classical form (type 1) includes progressive articular deformations, subcutaneous lumps, inflammatory granulomas, and progressive neurological dysfunction [16]. The aCDases from human [17] and mouse [18] have been cloned. The enzyme is localized to the lysosomes [19] or is secreted extracellularly [20]. 

The aCDase was shown to be upregulated in prostate cancer [21] and melanomas [22], which lead to the hypothesis that the aCDase could be a tumour marker [22, 23]. Moreover, aCDase in tumour cells obviously confers resistance to chemo- and radiotherapy [24] thus making the inhibition of this enzyme a potential target for cancer therapy [25].

**Figure 1:**
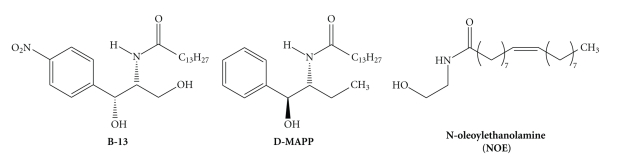
Current lead structures for the development of CDase inhibitors.

At present, there are three different lead structures for aCDase inhibitors (Figure 1). The aCDase is selectively inhibited by B-13 with an IC_50_ of about 10 *μ*M and less potently by N-oleoylethanolamine (NOE, *K*_*i*_ ~ 500 *μ*M). D-MAPP, which is structurally related to B-13, has been reported to selectively inhibit alkaline ceramidase [15] activity derived from HL-60 cell extracts with an IC_50_ of about 1–5 *μ*M [26]. In another report, however, D-MAPP is a moderate but selective inhibitor of aCDase in human melanoma and HaCaT cells [27]. 

The mechanism of ceramidase inhibition for all these substances is currently unknown. The three lead structures, B-13, NOE, and D-MAPP, can be obtained by a single synthetic step from commercially available chemicals [28], thus making them attractive starting points for combinatorial chemistry and SAR studies. 

**Figure 2:**
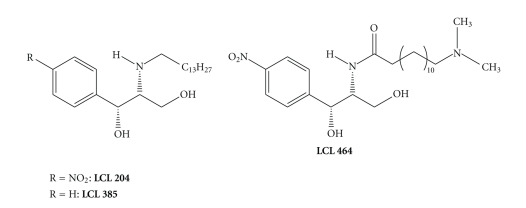
Some of the basic analogues of **D-MAPP **and **B-13** developed by Gatt et al. (LCL 204, LCL 385) and by Bielawska et al. (LCL 464).

Because of their potential as anticancer drugs, several novel ceramidase inhibitors have been developed recently. From a collection of NOE analogues, two compounds not only inhibited aCDase in cell culture (IC_50_ ~ 15 *μ*M), but at the same time exhibited cytotoxicity to A549 cells (IC_50_ ~ 40 *μ*M) [29, 30]. The screening of these potential CDase inhibitors has been facilitated by a fluorescent ceramidase probe allowing for high-throughput screening of CDase inhibitors *in vitro* and in cell culture [31]. In an effort to improve the activity of B-13 and D-MAPP *in vitro* and in cell culture, Bielawska and colleagues recently conducted several very detailed and interesting studies. In an attempt to develop aCDase inhibitors that accumulate in lysosomes, B-13 and D-MAPP analogues carrying basic functional groups have been evaluated (Figure 2) [32–34]. Several of these compounds were synthesized before in the group of *Shimon Gatt* [35, 36].

Similar to the tricyclic antidepressants imipramine and desipramine [37], these molecules are in equilibrium between the protonated cationic form and the unprotonated neutral form. Only the latter can permeate lysosomal membranes and enter the lysosomes. In the acidic environment of the lysosomes, these substances immediately get fully protonated. Very rapidly, as a consequence of the steady-state equilibrium, nearly the whole portion of the substance can be found in the lysosomes, thereby increasing its effective *in situ *concentration. 

Indeed, the substances LCL 204, LCL 385, and LCL 464 accumulated in the acidic compartments of the cells, while the neutral analogues showed no spatial preference. 

Interestingly, the lysosomotropic aCDase inhibitor LCL 204 was shown to induce apoptosis in prostate cancer cells [38] and to enhance the cytotoxicity of the viral protein apoptin in prostate cancer [39]. This compound is also active in head and neck squamous cell cancers (HNSCCs) *in vitro* and *in vivo* in a xenograft model sensitizing cells to Fas ligand-induced apoptosis [40]. In addition, the closely related analogue LCL-385, sensitized PPC-1 prostate cancer cells to radiation and significantly decreased tumor xenograft growth [41]. 

A more detailed biochemical investigation, however, revealed that LCL 204 (and probably also LCL 385) induce unwanted lysosomal permeabilization and degradation of aCDase [34]. A similar mode of action is known for the tricyclic antidepressants. These positively charged agents induce proteolytic degradation of acid sphingomyelinase and probably of acid ceramidase by blocking the negatively charged lipid bis(monoacylglycero)phosphate (BMP) [42], which is specific to the membranes of intralysosomal vesicles and has proven indispensable for the action of many sphingolipid hydrolases. Obviously, BMP promotes the binding of sphingolipid hydrolases and sphingolipid activator proteins (SAPs) to the intralysosomal membranes thereby protecting these proteins from proteolytic degradation. Recent investigation showed that BMP is essential for the binding of lysosomal Hsp70 which promotes lysosomal integrity [43]. Thus, agents comprising basic groups that may be protonated in the lysosomes have the potential to act by perturbing lysosomal integrity. In fact, similar to the tricyclic antidepressants, LCL 204 hardly inhibits aCDase from cell extracts (5% inhibition at 50 *μ*M). In contrast, B-13 (95%) is much more potent in cell extracts but revealed being virtually inactive against aCDase activity in intact MCF7 cells, probably due to its inability to accumulate in cellular lysosomes. The reason for the marked increase in cellular ceramide caused by B-13 is currently unknown. It has to be noted that in an earlier study, LCL 204 (AD2646) showed an IC_50_ of 42 *μ*M [44], underscoring the importance of using purified enzymes for SAR studies.

A second generation basic B-13 analogue, LCL 464, was shown to inhibit CDase activity in cell extracts (50% at 50 *μ*M). Compared to LCL 204 this compound is not only more potent in cellular extracts, but also shows superior inhibition of a CDase in living cells, without reducing the amount of aCDase at the protein level [34]. These results show that the cellular inhibition of acid ceramidase depends on both, the *in vitro* activity and the ability to accumulate in cellular lysosomes. The latter feature is dependent on the presence of basic moieties.

## 2. Results

**Figure 3:**
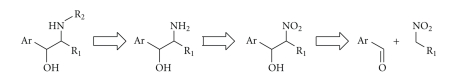
Retrosynthetic analysis of the B-13/D-MAPP scaffold.

The molecular basis of the ceramidase inhibition by D-MAPP and B-13 is still unknown. Accordingly, the possibilities for a rationallydriven synthesis of improved inhibitors are limited. Small libraries of compounds with systematically altered functional groups or stereochemistry can provide information about structural requirements for binding to ceramidases. Preceding the investigation of the structure-activity relationship (SAR) for B13-inhibiting acid ceramidase, we did a retrosynthetic analysis of the B-13/D-MAPP scaffold. It is known that the base of D-MAPP, norephedrine, can easily be obtained by nitroaldol reaction (“Henry reaction”) of nitroethane with benzaldehyde and subsequent reduction (see retrosynthetic analysis in Figure 3). 

**Figure 4:**
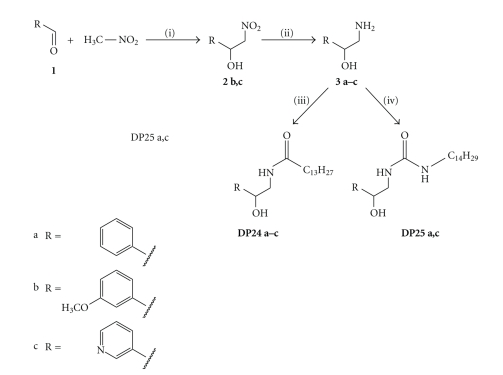
Synthetic route to the aCDase inhibitors **DP24 a–c** and **DP25 a,c**. Reagents and conditions: (i) triethylamine, r.t., 2–6 hrs; (ii) H_2_, Pd/C, methanol, r.t., 16 hrs; (iii) myristoyl chloride, pyridine, 4 hrs; (iv) tetradecyl isocyanate, acetonitrile/chloroform, r.t. 6 hrs.

**Figure 5:**
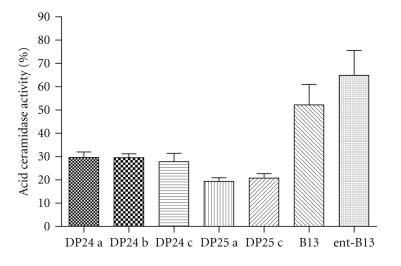
Activity of aCDase in the presence of 2.5 *μ*M of the novel analogues **DP24 a–c** and **DP25 a,c** as well as B13 and its enantiomer ent-B13. All experiments were conducted as triplicates and repeated at least once. *P*-values (two-tailed, in order of appearance):  .007,  .003,  .083,  .006,  .009,  .027,  .026.

**Figure 6:**
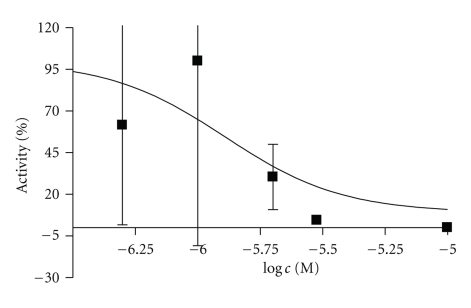
IC_50 _curve for DP24a. The IC_50_ value was calculated as being 1.287 *μ*M. Each concentration was measured in triplicate.

The retrosynthetic analysis revealed three variable moieties, an aromatic part (Ar), a nitrogen-bound substituent R_2_, and a nitrocomponent (O_2_N–CH_2_–R_1_). The simplest organic nitro component is nitromethane (R_1_ = H), leading to bases of type **3** (Figure 4). In our initial activity screening, the B-13 analogue **DP24a** displayed a remarkable inhibitory activity towards the recombinant human aCDase (Figure 5). In a more detailed study, the IC_50_ value was determined at 1.287 *μ*M (Figure 6).

The B-13 lead structure contains an aromatic nitro group which decreases electron density of the aromatic ring (−M effect). We wanted to test whether electron donors like methoxy groups (+M effect) would influence the inhibitory properties of our compounds. Reaction of 3-methoxy benzaldehyde **1b** with nitromethane yielded the nitrocompound **2b**. Catalytic hydrogenation of the latter afforded **3b**, which was treated with myristoyl chloride to give the final B-13 analogue **DP24b**. At a concentration of 2.5 *μ*M both, **DP24a** and **DP24b** showed superior inhibition of human aCDase, compared to B-13. These results suggest that neither the nitro- nor the methoxygroup influence the potency of aCDase inhibition. 

Next we wanted to evaluate alternative possibilities for an introduction of basic amino groups for a facilitating lysosomal enrichment. Lipid hydrolases obviously contain large hydrophobic pockets which harbour the fatty acid moieties of substrate lipids. Hydrophobic interaction however often is a critical determinate in drug design. Recently we have shown for three different substance classes inhibiting acid sphingomyelinase that the inhibitory potency positively correlates with the length of hydrocarbon chains [11–13]. We hypothesize that the remarkable drop in cell lysate aCDase inhibition from B-13 (95% inhibition) to LCL-464 (50% inhibition) is a result of the partial disruption of the major hydrophobic interaction between the aCDase and the positively charged fatty acid chain in LCL-464. This problem might be circumvented by introduction of basic moieties at sites other than the fatty acid chain. Thus we synthesized a pyridine analogue of **DP24** by reaction of nicotinic aldehyde **1c** with nitromethane. The resulting nitrocompound **2c** was reduced to the respective amine **3c** and reacted with myristoyl chloride to afford **DP24c**. Although **DP24c** provides a basic moiety, it showed at least the same inhibition as **DP24a** or **DP24b** (Figure 5).

Interestingly, we noticed that the enantiomer of B-13 (ent-B-13 comprising S, S stereochemistry) also showed a remarkable inhibition of a CDase, which is contradictory to the literature data obtained with cellular lysates. A possible explanation would be that ent-B-13 is readily cleaved by unknown enzymes from cellular lysates. 

Although early investigations did not provide evidence for B-13 being an alternative substrate to aCDase, we wanted to replace the biologically labile amide bond by the more stable urea group. Thus we synthesized the urea analogues **DP25a** and **DP25c** (Figure 4). Evaluation in the aCDase assay revealed only a slightly better inhibition, which is probably due to the slightly increased length of the hydrophobic tail of the B-13 analogues (Figure 5).

## 3. Conclusion

In our preliminary *in vitro* studies, we were able to identify a new B-13 analogue with increased potency versus the recombinant human aCDase. This new simplified compound can easily be synthesized and offers the possibility for a more diverse series of B-13 analogues. Moreover, we have proposed a new way for introducing weakly basic isosteres in the aromatic part of the molecule. Such a modification does not reduce *in vitro* inhibition of aCDase. In contrast, LCL 464 shows strongly reduced aCDase inhibition *in vitro* when compared to B-13 meaning that the high potency in cells is to a large extent due to lysosomal accumulation. However, it remains to be clarified whether the pyridine group of **DP24c** (p*K*_*b*_ ~ 9) is as suitable as the tertiary amino group of LCL 464 (p*K*_*b*_ ~ 3) for lysosomal targeting. Furthermore, it has to be noted that substances comprising a basic moiety for lysosomal enrichment may induce lysosomal permeabilization. Comparative cellular studies will be performed in the near future. 

In the course of our initial investigation of the structure-activity relationship of B-13 analogues, we abandoned the introduction of a defined stereochemistry at the asymmetric carbon atom. All the inhibition studies were done with racemic mixtures. Optical pure compounds, however, are possible via an enantioselective Henry reaction [45, 46].

## 4. Experimental

### 4.1. Experimental Details for the Synthesis of the Inhibitors

**General Procedure for the Synthesis of Henry Products 2b and 2c**

To a stirred solution of aldehyde **1b **or **1c** (1.5 mmol) in 10 mL nitromethane was added triethylamine (1 mL) as catalyst. The resulting solution was stirred at room temperature until the consumption of the aldehyde was indicated by TLC. It was diluted with ethyl acetate (200 mL), washed with sodium hydrogen sulfite, sat. sodium hydrogen carbonate, water, and brine, dried over anhydrous sodium sulfate, and concentrated in vacuum. The crude product was purified by column chromatography on silica gel (cyclohexane/ethyl acetate 2 : 1, *v/v*).

**General Procedure for the Reduction of Henry Products to Aminols 3b and 3c**

The Henry product (0.23 mmol) was dissolved in 5 mL methanol and charged with a catalytic amount of palladium on activated charcoal. The mixture was evaporated and aerated with hydrogen. The reaction mixture was stirred at room temperature until the consumption of the Henry products was indicated. The mixture was filtered through a pad of Celite and evaporated to dryness. The crude product was purified by column chromatography on silica gel (cyclohexane/ethyl acetate 1 : 1, *v/v*).

**General Procedure for the Preparation of Amide Inhibitors DP24 a–c**

A solution of aminol (0.145 mmol) in 5 mL pyridine was placed in an ice bath and treated with myristoyl chloride (0.143 mmol). The resulting solution was allowed to come to room temperature and stirred for 16 h. The reaction was evaporated to dryness. The crude product was dissolved and partitioned between dichloromethane and 1 M HCl, then washed with sat. sodium hydrogen carbonate, water, and brine, dried over anhydrous sodium sulfate, and concentrated in vacuum. The crude product was purified by column chromatography on silica gel (cyclohexane/ethyl acetate 8 : 1, *v/v*).

**General Procedure for the Preparation of Urea Inhibitors DP25 a,c**

To a stirred solution of aminol (0.188 mmol) in 5 mL acetonitrile and 5 mL chloroform was added tetradecyl isocyanate (0.376 mmol). The mixture was stirred at room temperature for 12 h. The reaction mixture was evaporated to dryness. The crude product was purified by column chromatography on silica gel (CH_2_Cl_2_/methanol 20 : 1, *v/v*).

**1-(3-Methoxyphenyl)-2-Nitroethanol 2b**

300 mg (2.21 mmol) aldehyde **1b** gave 355 mg (1.99 mmol, 90%) of a pale yellow liquid, C_9_H_11_NO_4_ (197 g/mol). **R**_**f**_ (ethyl acetate) = 0.2. **^1^H-NMR** (CDCl_3_; 300 MHz): *δ* = 2.87 (bs, 1H, OH), 3.83 (s, 3H, CH_3_), 4.51 (dd, *J*_1_ = 3.2 Hz, *J*_2_ = 13.3 Hz, 1H; CH_2_), 4.60 (dd, *J*_1_ = 9.4 Hz, *J*_2_ = 13.3 Hz, 1H, CH_2_), 5.43 (dd, *J*_1_ = 3.2 Hz, *J*_2_ = 9.4 Hz, 1H; CH), 6.90 (ddd, *J*_1_ = 1.1 Hz, *J*_2_ = 2.5 Hz, *J*_3_ = 8.3 Hz, 1H; CHarom.), 6.97 (m, 2H; CHarom), 7.32 (dd, *J*_1_ = 8.1 Hz, *J*_2_ = 8.1 Hz, 1H; CHarom) ppm. **^13^C-NMR** (CDCl_3_; 75 MHz): *δ* = 55.4, 70.9, 81.2, 111.5, 114.4, 118.1, 130.1, 139.8, 160.0 ppm. **HR-MS p ESI** calculated for C_9_H_11_NO_4_  − H^+^ m/z = 196.0609, found: 196.0615.

**2-Nitro-1-(Pyridin-3-yl)-Ethanol 2c**

200 mg (1.86 mmol) aldehyde **1c** gave 250 mg (1.48 mmol, 79.6%) of brown oil, C_7_H_8_N_2_O_3_ (170 g/mol). **R**_**f**_ (ethyl acetate) = 0.05. **^1^H-NMR** (CDCl_3_; 300,1 MHz): *δ* = 4.52 (dd, *J*_1_ = 3.6 Hz, *J*_2_ = 12.8 Hz, 1H, CH_2_), 4.61 (dd, *J*_1_ = 9.3 Hz, *J*_2_ = 12.8 Hz, 1H, CH_2_), 5.50 (dd, *J*_1_ = 3.6 Hz, *J*_2_ = 9.3 Hz, 1H, CH), 5.67 (bs, 1H, OH), 7.33 (ddd, *J*_1_ = 0.6 Hz, *J*_2_ = 4.9 Hz, *J*_3_ = 7.9 Hz, 1H, CHarom), 7.82 (td, *J*_1_ = 1.7 Hz, *J*_2_ = 1.7 Hz, *J*_3_ = 7.9 Hz, 1H, CHarom), 8.40 (dd, *J*_1_ = 1.6 Hz, *J*_2_ = 4.9 Hz, 1H, CHarom), 8.48 (d, *J* = 2.15 Hz, 1H, CHarom) ppm. **^13^C-NMR** (CDCl_3_; 75,4 MHz): *δ* = 68.4, 81.2, 124.2, 134.7, 135.4, 147.0, 149.1 ppm. **HR-MS p ESI** calculated for C_7_H_7_N_2_O_3_  − H^+^ m/z = 169.0608, found: 169.0615.

**1-(3-Methoxyphenyl)-2-Aminoethanol 3b**

170 mg (0.86 mmol) **2b** gave 124 mg (0.74 mmol, 86%) of a brown solid, C_9_H_13_NO_2_ (167 g/mol). **R**_**f**_ (CH_2_Cl_2_/methanol 10%) = 0.3. **^1^H-NMR** (CDCl_3_; 300 MHz): *δ* = 2.74–3.13 (m, 2H, CH_2_), 3.75 (s, 3H, CH_3_), 4.98 (d, *J* = 7.4 Hz, 1H; CH), 6.78 (dd, *J*_1_ = 2.1 Hz, *J*_2_ = 8.1 Hz, 1H; CHarom.), 6.89 (m, 2H; CHarom), 7.21 (dd, *J*_1_ = 7.9 Hz, *J*_2_ = 7.9 Hz, 1H; CHarom) ppm. **^13^C-NMR** (CDCl_3_; 75 MHz): *δ* = 49.0, 60.8, 74.0, 111.4, 113.0, 118.2, 129.5, 144.2, 159.7 ppm. **HR-MS p ESI** calculated for C_9_H_13_NO_2_  − H^+^ m/z = 168.1019, found: 168.1015.

**2-Amino-1-(Pyridin-3-yl)-Ethanol 3c**

132 mg (0.79 mmol) gave 109 mg (0.79 mmol, quant.) of a pale brown solid, which was used without purification, C_7_H_7_N_2_O_3_ (170 g/mol). **R**_**f**_ (CH_2_Cl_2_/methanol 10%) = 0.05.

**1-(2-Hydroxy-2-Phenylethyl)-3-Tetradecylurea DP25a**

100 mg (0.73 mmol) 1-phenyl-2-aminoethanol gave 150 mg (0.40 mmol, 54.5%) of a colorless solid, C_23_H_40_N_2_O_2_ (376 g/mol). **R**_**f**_ (CH_2_Cl_2_/methanol 10%) = 0.5. **^1^H-NMR** (CDCl_3_; 300 MHz): *δ* = 0.90 (t, 3 H, CH_3_), 1.29 (s, 22H, CH_2_), 3.13 (dd, *J*_1_ = 7.1 Hz, *J*_2_ = 7.1 Hz, 2H, NCH_2_), 3.31 (dd, *J*_1_ = 7.6 Hz, *J*_2_ = 14.4 Hz, 1H, CH), 5.67 (bs, 1H, OH), 3.55 (dd, *J*_1_ = 2.8 Hz, *J*_2_ = 14.4 Hz, *J*_3_ = 7.9 Hz, 1H, CHarom), 4.83 (dd, *J*_1_ = 2.7 Hz, *J*_2_ = 7.5 Hz, 1H, CHarom), 5.50 (1H, NHurea), 5.80 (1H, NHurea), 7.28–7.30 (m, 5H, CHarom) ppm. **^13^CNMR** (CDCl_3_; 75 MHz): *δ* = 14.1, 22.7, 26.9, 29.4, 29.6, 29.7, 30.1, 31.9, 40.8, 48.7, 74.7, 125.9, 127.6, 128.4, 142.2 ppm. **HR-MS p ESI** calculated for C_23_H_40_N_2_O_2_ + H^+^ m/z = 377.3163, found: 377.3163.

**1-(2-Hydroxy-2-(Pyridin-3-yl)Ethyl)-3-Tetradecylurea DP25c**

20 mg (0.14 mmol) crude 1-(pyridine-3-yl)-2-aminoethanol gave 15 mg (0.040 mmol, 28.4%) of a colorless solid, C_22_H_39_N_3_O_2_ (377 g/mol). **R**_**f**_ (CH_2_Cl_2_/methanol 10%) = 0.2. **^1^H-NMR** (CDCl_3_; 300 MHz): *δ* = 0.87 (t, 3 H, CH_3_), 1.24 (s, 22H, CH_2_), 1.45 (br, 2H, CH_2_), 2.85 (br, 2H, NCH_2_), 3.12 (br, 1H, OH) 3.30–3.38 (br, 1H, CH_2_), 3.60 (d, *J* = 15.4 Hz, 1H, CH_2_), 4.79 (br, 1H, NHurea), 4.89 (dd, *J*_1_ = 2.5 Hz, *J*_2_ = 6.9 Hz, 1H, CH), 5.07 (br, 1H, NHurea), 7.31 (dd, *J*_1_ = 3.7 Hz, *J*_2_ = 8.3 Hz, 1H, CHarom), 7.80 (d, *J* = 8.5 Hz, 1H, CHarom), 8.48 (br, 1H, CHarom), 8.57 (br, 1H, CHarom) ppm. **^13^C-NMR** (CDCl_3_; 75 MHz): *δ* = 14.1, 22.8, 27.0, 29.4, 29.7, 29.8, 30.2, 32.0, 40.3, 47.8, 71.7, 123.9, 134.9, 138.7, 147.1, 148.0, 160.2 ppm. **HR-MS p ESI** calculated for C_22_H_39_N_3_O_2_ + H^+^ m/z = 378.3115, found: 378.3118.

**N-(2-Hydroxy-2-Phenylethyl)-Tetradecanamide DP24a**

100 mg (0.73 mmol) 1-phenyl-2-aminoethanol gave 118 mg (0.34 mmol, 46.6%) of a colorless solid, C_22_H_37_NO_2_ (347 g/mol). **R**_**f**_ (CH_2_Cl_2_/methanol 10%) = 0.6 **^1^H-NMR** (CDCl_3_; 300 MHz): *δ* = 0.90 (t, 3 H, CH_3_), 1.28 (s, 20H, CH_2_), 1.57 (br, 2H, CH_2_), 2.11 (t, 2H, NCH_2_), 3.27 (m, 1H, CH_2_), 3.61 (ddd, *J*_1_ = 3.2 Hz, *J*_2_ = 6.7 Hz, *J*_3_ = 13.9 Hz, 1H, CH_2_), 4.77 (dd, *J*_1_ = 3.1 Hz, *J*_2_ = 7.9 Hz, 1H, CH), 6.48 (br, 1H, NHamide), 7.31 (m, 5H, CHarom) ppm. **^13^C-NMR** (CDCl_3_; 75 MHz): *δ* = 14.1, 22.7, 25.7, 29.3, 29.4, 29.5, 29.7, 31.9, 36.6, 47.8, 73.5, 125.8, 127.7, 128.4, 141.9, 174.9 ppm. **HR-MS p ESI** calculated for C_22_H_37_NO_2_ + H^+^ m/z = 348.2897, found: 348.2897.

**N-(2-Hydroxy-2-(3-Methoxyphenyl)Ethyl)-Tetradecanamide DP24b**

15 mg (0.090 mmol) 1-(3-Methoxyphenyl)-2-aminoethanol gave 20 mg (0.053 mmol, 58.9% d. Th.) of a white solid, C_23_H_39_NO_4_ (377 g/mol). **R**_**f**_ (CH_2_Cl_2_/methanol 10%) = 0.5. **^1^H-NMR** (CDCl_3_; 300 MHz): *δ* = 0.90 (t, 3 H, CH_3_), 1.27 (s, 20H, CH_2_), 1.62 (br, 2H, CH_2_), 2.20 (dd, *J*_1_ = 7.6 Hz, *J*_2_ = 15.2 Hz, 1H, NCH_2_), 2.34 (m, 1H, NCH_2_), 3.82 (s, 3H, OCH_3_) 3.87 (d, *J* = 8.0 Hz, 1H, CH), 6.00 (br, 1H, NHamide), 6.85–7.00 (m, 3H, CHarom) 7.25–7.32 (m, 1H, CHarom) ppm. **^13^C-NMR** (CDCl_3_; 75 MHz): *δ* = 14.1, 22.7, 25.7, 29.1, 29.3, 29.4, 29.5, 29.7, 31.9, 36.6, 55.2, 73.7, 111.2, 113.3, 118.1, 129.5, 159.8 ppm. **HR-MS p ESI** calculated for C_23_H_39_NO_3_ + H^+^ m/z = 378.3003, found: 378.3002.

**N-(2-Hydroxy-2-(Pyridin-3-yl)-Ethyl)Tetradecanamide DP24c**

20.5 mg (0.15 mmol) crude 1-(pyridine-3-yl)-2-aminoethanol gave 37 mg (0.106 mmol, 71.6%) of a colorless solid, C_21_H_36_N_2_O_2_ (348 g/mol). **R**_**f**_ (CH_2_Cl_2_/methanol 10%) = 0.4. **^1^H-NMR** (CDCl_3_; 300 MHz): *δ* = 0.87 (t, 3 H, CH_3_), 1.27 (s, 20H, CH_2_), 1.58 (m, 2H, CH_2_), 2.20 (m, 2H, COCH_2_), 3.12 (br, 1H, OH) 3.40 (dd, *J*_1_ = 7.1 Hz, *J*_2_ = 14.1 Hz, 1H, CH_2_), 3.69 (ddd, *J*_1_ = 3.1 Hz, *J*_2_ = 6.4 Hz, *J*_3_ = 14.2 Hz, 1H, CH_2_), 4.91 (dd, *J*_1_ = 2.6 Hz, *J*_2_ = 7.0 Hz, 1H, CH), 5.26 (br, 1H, NHamide), 7.31 (dd, *J*_1_ = 5.0 Hz, *J*_2_ = 7.4 Hz, 1H, CHarom), 7.78 (d, *J* = 7.9 Hz, 1H, CHarom), 8.48 (br, 1H, CHarom), 8.54 (br, 1H, CHarom) ppm. **^13^C-NMR** (CDCl_3_; 75 MHz): *δ* = 14.1, 22.7, 25.7, 29.3, 29.4, 29.5, 29.7, 31.9, 36.5, 47.6, 71.8, 123.7, 134.4, 147.2, 148.4, 175.4 ppm. **HR-MS p ESI** calculated for C_21_H_36_N_2_O_2_ + H^+^ m/z = 349.2850, found: 349.2852.

### 4.2. Micellar aCDase Assay

The micellar aCDase assay has been described previously [47]. 

Briefly, assays were performed in the presence of sodium acetate buffer (250 mM, pH 4.0), 5 mM EDTA, 0.7% (w/v) Triton X-100, 0.4% (w/v) Tween 20, 0.4% (w/v) Igepal CA-630, and 17 nmol/50 *μ*L N-Lauroyl-D-*Erythro*-Sphingosine. The activity was determined with 60 *μ*L of buffer, 35 *μ*L undiluted enzyme solution (43,7 *μ*g/mL), and 5 *μ*L of 50 *μ*M inhibitor solution in DMSO which gave 100 *μ*L total volume with 2.5 *μ*M inhibitor concentration. Tests were incubated for 30 min at 37°C, the reactions terminated with 800 *μ*l chloroform/methanol 2 : 1 (v/v) and 200 *μ*L saturated aqueous ammonium carbonate solution, and C_14_- and C_16_-sphinganin in methanol (500 pM of each) were added. Afterwards the mixtures were centrifuged at 10000 × g for 5 min, the chloroform phase was dried and redissolved in 50 *μ*L ethanol and incubated 10 min at 50°C. 50 *μ*L o-phthalaldehyde solution was added. The mixture was incubated for 5 min at room temperature. Samples were diluted with 0.9 mL HPLC buffer (acetonitrile/2 mM KH_2_PO_4_, pH 7.0, 9/1 (v/v)) and 0.1 mL 5 mM KH_2_PO_4_ buffer (pH 7.0, 90 : 10, v/v). Aliquots were injected immediately onto an RP-C18 Polaris A 5 *μ*m (PN A 2000-250×100)—column by Varian. Lipids were eluted with mixtures out of water containing 1% acetonitrile and acetonitrile containing 1% water with a flow of 1 mL/min. Excitation wavelength is *λ* = 355 nm, emission wavelength is *λ* = 435 nm. For IC_50_ determination, the inhibitor concentrations were varied as indicated. All experiments were done in triplicate.
